# Streamlining the Highly Reproducible Fabrication of Fibrous Biomedical Specimens toward Standardization and High Throughput

**DOI:** 10.1002/adhm.202402527

**Published:** 2024-12-15

**Authors:** Zan Lamberger, Camilla Mussoni, Nicoletta Murenu, Mateo Andrade Mier, Philipp Stahlhut, Taufiq Ahmad, Natascha Schaefer, Carmen Villmann, Sarah Zwingelberg, Jürgen Groll, Gregor Lang

**Affiliations:** ^1^ Department of Functional Materials in Medicine and Dentistry and Bavarian Polymer Institute University of Würzburg Pleicherwall 2 D‐97070 Würzburg Germany; ^2^ Institute for Clinical Neurobiology University Hospital Würzburg Versbacherstr. 5 D‐97078 Würzburg Germany; ^3^ Department of Ophthalmology University Hospital Düsseldorf Merowingerplatz 1A 40225 Düsseldorf Germany

**Keywords:** electrospinning, fibers, melt‐electrowriting, standardization, upscale

## Abstract

Soft nano‐ and microfiber‐based polymer scaffolds bear enormous potential for their use in cell culture and tissue engineering since they mimic natural collagen structures and may thus serve as biomimetic adhesive substrates. They have, however, so far been restricted to small‐scale production in research labs with high batch‐to‐batch variation. They are commonly produced via electrospinning or melt electrowriting and their delicate nature poses obstacles in detachment, storage, and transportation. This study focuses on overcoming challenges in the high throughput production and practical handling, introducing new methods to reproducibly prepare such scaffolds suitable for quantitative cell culture applications. Attention is given to the seamless handling and transfer of samples without compromising structural integrity. Challenges in detaching fibers without damage as well as storage, and transport are addressed. Cell culture studies demonstrate the methodological advantages, emphasizing the potential for standardized testing and biological readouts of these delicate fiber materials. The developed methods are applicable across various electrospinning and melt electrowriting approaches and can essentially contribute to their utilization in laboratory research and commercial applications.

## Graphical abstract

Biopolymer‐based nano‐ and microfiber scaffolds can be designed to mimic collagen structures, rendering them highly attractive for, e.g., regenerative medicine. However, their production is often limited to small scales with high variability. This study presents a robust high‐throughput method for the standardized preparation of such delicate scaffolds, ensuring reproducibility, structural integrity, and adaptability for both laboratory work and commercial biomedical applications.

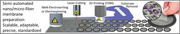

## 1 Introduction

Electrostatic spinning methods, including electrospinning (ES) and melt electro‐writing (MEW), have gained considerable attention in scientific research in recent decades. Their capability to create fiber diameters in the nano‐ and submicron ranges, along with the extensive diversity of synthetic and natural polymers available for use renders them particularly suitable for tissue engineering.^[^ ^1^, ^2^ ^]^ These methods facilitate the fabrication of soft biomimetic scaffolds resembling the fibrillar collagen structures present in natural tissues (50–500 nm).^[^ ^3^, ^4^ ^]^ Furthermore, ongoing research investigates their potential in other medical applications such as wound coverings,^[^ ^5^ ^]^ drug release,^[^ ^6^ ^]^ as well as in the development of lab‐ and organ‐on‐a‐chip devices.^[^ ^7^, ^8^ ^]^ Additionally, electrostatic spinning methods prove highly suitable for diverse technical applications due to the high surface‐to‐volume ratio of thin fibers and their remarkable porosity. These applications span various fields, including filtration,^[^ ^9^ ^]^ textiles,^[^ ^10^ ^]^ and sensing.^[^ ^11^, ^12^ ^]^ Despite their substantial potential, notably in the pharmaceutical sector, the commercial implementation of these material systems remains limited.^[^ ^13^, ^14^ ^]^ However, it is anticipated that with advancing technological developments, their market for laboratory research and industrial production will witness substantial growth.^[^ ^15^ ^]^ A recent review article by Ji et al. underscores the urgent need to devise innovative methods for scaling up nano‐/micro‐fiber materials while simultaneously preserving their quality and consistency.^[^ ^16^ ^]^ Nadaf et al. also highlighted this crucial aspect, emphasizing that stability is the primary challenge when dealing with nanofibers.^[^ ^17^ ^]^ Concurrently, there is a strong call to establish standards in processing and testing.

The electrostatic spinning process itself is relatively simple, requiring only a high‐voltage source, a needle pump, a collector, and a suitable polymer solution or melt. Unlike thicker mats, which are easier to handle and often used for filters or wound dressings,^[^ ^18^ ^]^ processing delicate fiber mats poses a significant challenge, especially if they are not deposited directly onto the target.^[^ ^19^ ^]^ In such cases, spinning typically occurs directly onto a grounded or oppositely charged collector plate, or, for aligned fibers, onto a rotating cylinder. Subsequently, the fiber mats must be carefully peeled off for further preparation, a step that may lead to damage such as cracks, holes, or overstretching of the nonwoven membrane. In many applications, the fibers need to undergo chemical modifications or coatings for functionalization.^[^ ^13^, ^20^, ^21^, ^22^, ^23^, ^24^ ^]^ This necessitates the transfer of samples into various agents, introducing mechanical stress and potential damage. With the advancement of increasingly precise analytical methods, such as those for characterizing the surfaces of functionalized fibers, the demands for the quality of the required samples also increase.^[^ ^25^ ^]^ Correlative methods, for example, require the examination of a single sample in various devices, where selected spots must be precisely locatable. Wang et al. identified sample preparation as the greatest challenge in this regard and concluded, that especially in the field of biomaterials, novel fixation procedures need to be developed to better preserve cell and material structures.^[^ ^26^ ^]^ In particular, thin nanofiber mats tend to collapse and entangle in a humid state, resulting in deformation and damage. Often, samples must be cut to an appropriate size for further testing or application, a process that can also compromise sensitive structures. In the past, individual solutions have been applied to address these challenges, such as detaching the nonwovens submersed in ethanol/water mixtures, low adhesive substrates or utilizing coatings such as gelatine.^[^ ^27^, ^28^, ^29^, ^30^, ^31^ ^]^ Additionally, the fabrication of thicker fiber mats has been employed to enhance handling. Inserts have been used to immobilize samples, although this can lead to damage during the clamping of the nonwovens and, like the other strategies is hardly scalable. Pensa et al. used 3D printing to reinforce electrospun scaffolds by depositing a mesh onto the electrospun mat.^[^ ^32^ ^]^ Liu et al. electrospun fibers on top of a 3D‐printed construct to produce scaffolds for heart‐on‐a‐chip applications.^[^ ^33^ ^]^ In both approaches, electrospun fiber mats were reinforced across their entire surface, which is suitable for the applications described. However, this can lead to limitations in many cases, for example, when the fiber mats are open‐pored or colonized on both sides. In these cases, the support structures hinder the growth and migration of cells. Jin et al. used a melt‐electrowriting machine to precisely adjust the mechanical properties of their scaffolds through a combination of thick and thin strands.^[^ ^34^ ^]^ However, this elegant method is only suitable for melt‐electrowriting and can only be implemented with specialized equipment. Since electrospinning produces smaller fibers and the nanofibrous structures are highly attractive, e.g., in the field of skin tissue engineering, and a universally applicable workflow to ensure the quality and integrity of soft nanofiber mats for various processing and application possibilities while consistently and adequately preparing samples in sufficient quantities is of high interest. Frequently, the decision must be made between expedited handling that may cause damage to the scaffolds and laborious, slow work aimed at preserving the structures. This choice has significant implications for the reproducibility of results.

Based on recurring issues and limitations in fabricating and applying such sensitive samples, we formulated key design criteria for a widely applicable process:

- Efficient membrane handling: Enables preparation, immobilization, and transfer of sensitive nano‐/microfiber membranes.
- Versatile application: Compatible with diverse materials, methods, and fiber morphologies without reparameterization.
- Accessible technology: Utilizes standard lab equipment for ease of use.
- Semi‐automation: Minimizes operator influence, ensuring reproducibility.
- Design flexibility: Adapts to various bioreactors and well‐plate sizes.
- Scalable production: High‐throughput, reproducible sample generation with minimal personnel input.

To address these requirements associated with handling sensitive electrospun and melt‐electrowritten samples in regenerative medicine, we developed a new workflow that is not only easily implementable, universally applicable, scalable, and cost‐effective but can also be carried out with standard laboratory equipment or minimal investment. This workflow employs a sacrificial film as a substrate material, combined with laser cutting and fused deposition modeling (FDM), enabling high‐throughput production of delicate scaffolds. It ensures that the scaffolds remain manageable for virtually any subsequent step, whether it is functionalization, analytical or application‐oriented, while maintaining their structural integrity. By providing a semi‐automated and reproducible process, this method provides the careful handling of these fragile materials, which is crucial for their use in tissue engineering and other regenerative medicine applications. Additionally, the scalability of the method allows for the efficient production of large quantities of scaffolds, facilitating both research consistency and clinical application.

In summary, this work provides a transformative solution for preparing and managing sensitive nano‐ and microfiber‐based materials, with the potential to accelerate advancements in regenerative medicine, tissue engineering, and beyond. By offering a method that is not only technically sound but also scalable, cost‐effective, and easy to implement, it paves the way for more consistent, high‐quality research and standardization enabling faster translation from laboratory to, e.g., clinic.

## 2 Results

### 2.1 Basic Workflow to Harvest Electrospun and Electrowritten Scaffolds

**Figure 1:**
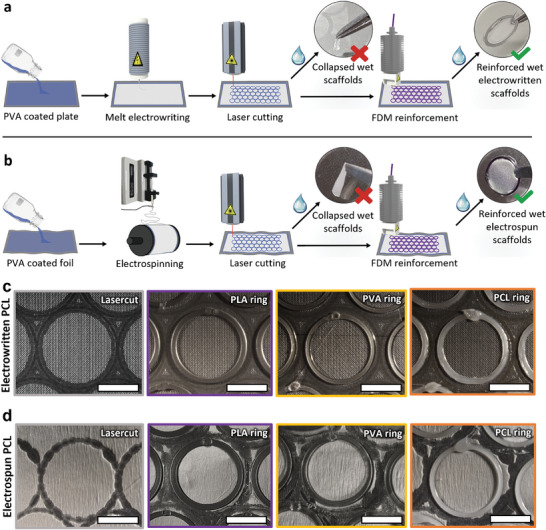
Basic concept of scaffold processing. Schematic drawing showing the new workflow used in conjunction with a) melt‐electrowritten scaffolds and b) electrospun fiber mats. c) Melt‐electrowritten scaffolds and d) electrospun fiber mats after laser cutting, as well as with FDM printed support rings made of PLA, PVA, and PCL. Scale bar for (c,d) corresponds to 5 mm.

To facilitate seamless removal of the electrospun or electrowritten scaffolds from their substrates after production, we used a water/ethanol soluble sacrificial cast PVA coating cast on steel plates or aluminum foil. The FDA‐approved PVA is known to not adsorb to polymers that constitute the scaffolds, whilst also being inert for cells and biocompatible (**Figure** **1**a,b).^[^ ^35^, ^36^ ^]^ Moreover, when combined with laser cutting, which is practical for quick cutting and scaling up substrate production, PVA plays a crucial role in ensuring seamless removal from the substrate. Without PVA, the polymer fuses to the substrate at the cut edges. The coating functions as a separation, which is then dissolved, and the scaffold released, also offering the advantage of combining the washing steps and sterilization for cell culture if done in 70% ethanol.

The issue of scaffold collapse, resulting from the weight of the liquid or surface tension upon wetting, causing deformation or entanglement, was addressed by employing FDM 3D printing reinforcements along the edges of the scaffolds (Figure 1a,b). These reinforcements kept the scaffolds taut when wetted, providing a stable point for handling without direct contact with the scaffold itself. The deposited polymer also fused the edges of the substrate together, further stabilizing substrates prone to delamination. The polymers used for printing the reinforcements were PCL, PLA and PVA (Figure 1c,d and Figure S1, Supporting Information), all FDA‐approved, biocompatible and non‐cytotoxic, rendering them ideal materials for cell culture applications.^[^ ^37^, ^38^, ^39^ ^]^ The polymers adhered to the substrates without heating the 3D printer build plate, rendering compromises between 3D printer bed temperatures and scaffold integrity due to heating obsolete. Moreover, by adding glycerin to the PVA coating, the adhesion of the produced scaffold and FDM strut could be even further improved. Within this process, virtually any printable shapes and sizes of FDM reinforcements are possible. While we predominantly showcased single‐layer circular reinforcements here, multi‐layer high reinforcements are also applicable. Moreover, entire 3D structures, like components for bioreactors or inserts, can be directly printed onto the substrates and subsequently detached by dissolving the PVA base.

### 2.2 Investigation of Potentially Critical Aspects of the New Procedure

Several experiments were conducted to critically assess potential issues in the new procedure. Firstly, the impact of temperatures during laser cutting and FDM printing on the delicate structure of the scaffolds was examined. Additionally, it was verified whether traces of the sacrificial layer made of PVA/Glycerin were detectable after detaching and washing the scaffolds. The mechanical resilience of the ring‐reinforced fiber samples was evaluated through tensile tests and a practical bending test. Lastly, the question was addressed as to whether the method can be transferred to other materials. The results are presented below.

#### 2.2.1 Impact of Temperature

**Figure 2:**
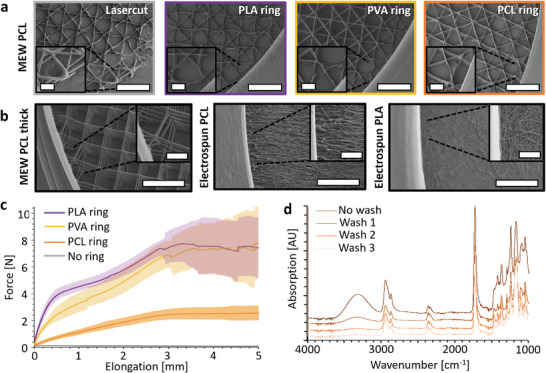
Detailed analysis of reinforced scaffolds. a) SEM images of electrowritten scaffolds after laser cutting, and reinforcement with PLA, PVA or PCL rings at the interface of the rings with the scaffolds. b) Various other scaffolds made of either PCL or PLA reinforced with PLA rings. c) Tensile tests of the different reinforcement rings and the thin MEW scaffold from a without rings. d) FTIR spectra showing the removal of the PVA from the MEW scaffolds after repeated washing steps in 70% ethanol as indicated by the stepwise decrease of the PVA‐characteristic broad O─H stretching band (3685–3010 cm^−1^).^[^ ^40^ ^]^ Scale bar a 400 µm,100 µm on zoomed in images, for b 400 µm on large pictures, on zoomed images 100 µm, 100 µm and 40 µm from left to right.

An important aspect to be considered in FDM printing on fiber constructs is the influence of heat during the extrusion of the melt. For instance, PLA and PVA have significantly higher melting points and, consequently, extrusion temperatures compared to the melt temperature of the PCL scaffolds produced here. To assess whether the delicate PCL structures are damaged by the radiant heat from the nozzle or the molten strand during the printing of support rings, a highly sensitive 20‐layer PCL MEW scaffold with 4 µm fiber diameters was supported with PLA (printing temperature 180 °C), PVA (printing temperature 190 °C), and PCL (printing temperature 130 °C). The transitions from the ring to the scaffold were then analyzed using SEM (**Figure** **2**a). It was observed that the most significant influence occurs already during laser cutting, where the edges fuse due to heat radiation of the laser spot. Nevertheless, this is not a critical issue, as these regions will later be incorporated into the support ring. Reasonably less was seen on the edges of the PLA and PVA reinforced scaffolds, while with the PCL reinforcement, there was no trace thereof due to the lower printing temperature. The effects of excess heat damaging the scaffolds were generally minimal, only affecting the edges of the scaffolds if at all.

#### 2.2.2 Material Variations

To assess the general applicability of the method, several different scaffolds of various materials and characteristics were processed (Figure 2b and Figure S2a, Supporting Information). This consistently worked, except when combining PLA scaffolds with PCL reinforcement rings, as these tendentially separated from the scaffold, due to too low printing temperatures to bond the materials.

#### 2.2.3 Mechanical Impact

An important objective of the method is to make the samples resistant to mechanical influences during handling. To verify this, tensile tests and bending tests were conducted with various samples. As anticipated, the reinforcement samples exhibited distinct behavior under tensile stress (Figure 2c), with PCL being more flexible, while PLA and PVA were stiffer. Importantly, it was evident that the reinforced samples were highly resistant to deformation. Moreover, the reinforcement ring or the fiber scaffold would tear apart rather than separating from each other, as they were sufficiently fused (Figure S2b, Supporting Information). To simulate the practical scenario of transferring a wet sample, such as grasping it with tweezers, the samples underwent a semi‐quantitative hanging test when dry and wetted. In this test, a straight scaffold would have a hanging angle of 0°, while a completely collapsed scaffold would measure an angle of 90° (Figure S3a, Supporting Information). The dry scaffolds were straight and could support their own weight, whereas the unreinforced ones remained slightly misshapen (Figure S3b, Supporting Information). Upon wetting, only the reinforced scaffolds maintained their hanging angle of 0°, while all others bent or completely collapsed, resulting in a change in angle. The varied hanging angles were influenced by factors such as fiber diameters, geometries, layer‐to‐layer adhesion, thickness, porosity, material, etc., indicating a high dependence on scaffold type for deformation and handleability. In contrast, the effects of inherent scaffold properties were nullified, and their stability significantly improved when reinforced with FDM.

#### 2.2.4 Residual Sacrificial Polymer

The sacrificial PVA coating enables seamless detachment of the scaffolds from the substrates even in 70% ethanol. Thereafter the removal of the PVA residues from the scaffold is an important aspect, which was investigated using FTIR (Figure 2d and Figure S2c, Supporting Information), showing that after two washing steps any measurable traces of PVA were removed from electrowritten and electrospun scaffolds.

### 2.3 Application of the Method to Challenging Scaffolds

To demonstrate the versatile applicability of our procedure, we addressed several common challenges encountered when working with soft nanofiber‐based materials: high‐throughput scaffolds for quantitative screenings, super‐fine scaffolds, and scaffolds for accurate placement.

#### 2.3.1 High Throughput Scaffolds

**Figure 3:**
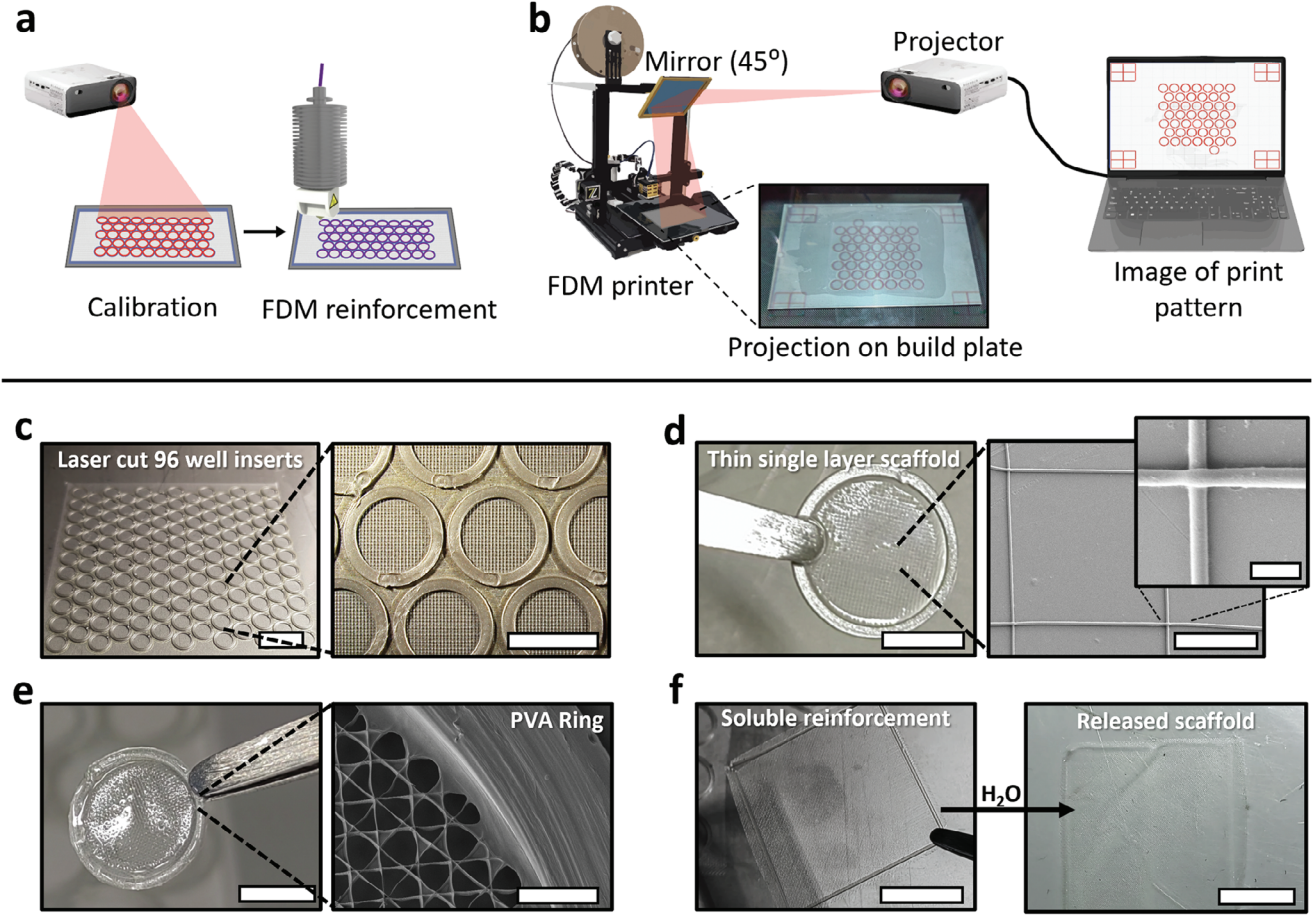
Further improvements and capabilities of reinforced scaffolds. a) Schematic showing calibrating the printed pattern using a projection before FDM printing. b) Projection calibration setup for the FDM printer, when using substrates that are not processed on the same device. c) Image of a laser cut and reinforced scaffold with an appropriate size for 96 well plates, showing the precision that can be achieved using the projection calibration setup. d) A wetted single‐layer MEW box scaffold suspended in the air, with the corresponding SEM images, showing the stability the reinforcement rings can provide even the most fragile unhandleable scaffolds. e) A MEW scaffold with a PVA reinforcement ring after dissolution of the PVA base layer and subsequent immersion in isopropanol to stop further dissolution of the reinforcement. f) A square PVA reinforced MEW scaffold being released from the reinforcement after dissolution of the ring in aqueous media. Scale bar c) 5 mm in magnification, d) 5 mm, 100 µm and 10 µm on magnifications, e) 200 µm and f) 1 cm.

Using a MEW scaffold, it was shown that, through automated laser cutting in combination with FDM printing, 137 precisely identical samples suitable for a 96‐well plate (**Figure** **3**c) could be processed within a short time (< 30 min) and with minimal effort. The material loss in this method is minimal, as almost the entire fiber mat can be utilized. Furthermore, an add‐on method was developed to expedite the calibration step in FDM (Figure 3a) for complex sample geometries or frequently altered geometries. In this approach, a projector is employed, which can project onto the built plate of the printer using a mirror (Figure 3b and Figure S4, Supporting Information). Initially, a calibration was printed onto the build plate, the projection aligned with it, and the print was removed. Subsequently, the precise print location could be projected onto the laser‐cut substrate when placed on the build plate. This feature makes it particularly suitable for multistep processing, as none of the preceding steps need to be calibrated or precise, thereby nullifying any cumulative errors that would otherwise be introduced by each processing step.

#### 2.3.2 Super Fine Scaffolds

The utilization of advanced processing techniques allows for the handling of extremely thin scaffolds, exemplified by a one‐layer box‐shaped MEW PCL scaffold with a square edge length of 200 µm and a fiber diameter of 4 µm. This scaffold, suspended in the air and wetted at a 1 cm reinforcement ring diameter (Figure 3d), becomes feasible through the incorporation of reinforcement and PVA‐facilitated removal. This approach extends to other thin and delicate scaffolds, provided that the scaffold material can adequately support its own weight and the surrounding liquid without undergoing deformation.

#### 2.3.3 Scaffolds for Accurate Placement

In some applications, such as with implants, it may be necessary for a nanofiber construct to be precisely placed and sutured, e.g., on native tissue. In such a case, it is essential to prevent the construct from collapsing or being damaged in a moist environment. While a reinforcing frame can prevent this, it may not be desired on the implant since it reduces its mechanical flexibility to adapt to organic topography and deformation. To demonstrate the suitability of the method for such applications, a reinforcing frame was 3D printed using PVA. It was shown that the dissolution of the PVA sacrificial coating to remove the scaffolds did not compromise the PVA reinforcement frame since it dissolves at a slower rate, and after detachment, further dissolution of the frame can be halted by immersion in isopropanol, an antisolvent (Figure 3e). PVA reinforcement may hence be used to stabilize the scaffold during handling and modification in non‐aqueous media and maybe even assist whilst suturing scaffolds. This may be relevant for wound coverings,^[^ ^41^, ^42^, ^43^ ^]^ heart patches or other implants,^[^ ^44^, ^45^ ^]^ where the biocompatible and low‐cytotoxic frame could later dissolve in aqueous media (Figure 3f) or body fluids. Strikingly, the method could also be transferred to other selectively water‐soluble polymers such as polyoxazolines or even tissue adhesive materials.^[^ ^46^, ^47^ ^]^

### 2.4 Two Illustrative Case Studies: Standard Cell Culture and Bioreactor Application

To demonstrate the added value of the new method, two case studies were conducted and documented. First, highly sensitive samples were tested in a standard cell culture setting (in vitro test in well plates). In a second example, samples were prepared specifically for use in a bioreactor and subjected to treatment according to a representative protocol. The results of both case studies are presented below.

#### 2.4.1 Standard Cell Culture

**Figure 4:**
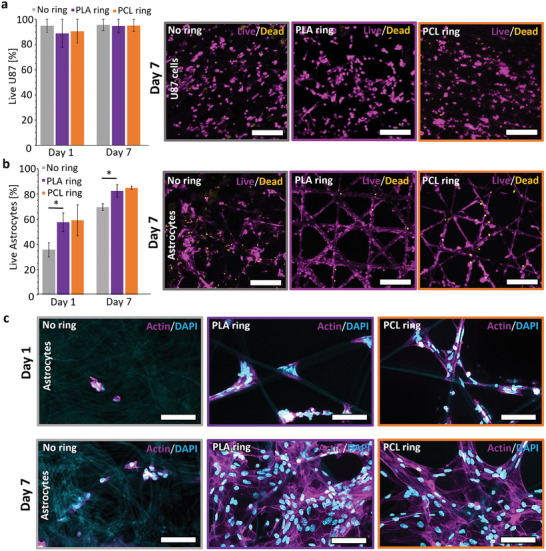
Cell cultural evaluation of reinforced and unreinforced scaffolds. a) Live/dead on days 1 and 7 of U87 cells on aligned electrospun PCL scaffolds with and without PCL/PLA reinforcement rings with representative images of each condition on day 7. b) Live/dead on days 1 and 7 of Astrocytes on MEW scaffolds unreinforced or reinforced with PCL/PLA rings, with representative images of day 7. c) Images of Actin/DAPI staining depict form‐stable reinforced scaffolds alongside misshapen unreinforced scaffolds, captured on both day 1 and day 7 under identical conditions as described in section B. The images also illustrate the scaffold geometry through DAPI staining. Plotted values in a and b presented as mean ± live/dead ratio, *n* = 3, three repetitions, *p*‐values were calculated using a one‐way ANOVA, *p < 0.05. Scale bar: a) 100 µm, b) 200 µm and in c) 50 µm.

To assess the impact of FDM‐printed reinforcements on cell culture outcomes, we compared ring‐shaped scaffolds reinforced with PCL and PLA to their unreinforced counterparts. Initially, these scaffolds were evaluated alongside aligned electrospun PCL membranes using U87 murine glioblastoma cells, which are relatively insensitive.^[^ ^48^, ^49^, ^50^ ^]^ Live/dead analysis revealed that the reinforcements did not significantly affect cell behavior, as cells performed similarly on all scaffolds (**Figures** **4**a and S5a, Supporting Information). The primary enhancement with the reinforcements was improved handleability, streamlining storage, removal, sterilization, and washing processes. The taut scaffold facilitated faster and easier manipulation, allowing for efficient transfer using tweezers.

Since scaffold geometry influences cells, we assessed the impact of reinforcements on deformation‐prone scaffolds utilizing thin MEW PCL scaffolds with triangular patterns.^[^ ^51^ ^]^ These scaffolds were chosen due to their susceptibility to delamination and overall difficulty in handling, attributed to low layer‐to‐layer adhesion and thin fiber diameters. Murine Astrocytes, known for their sensitivity and preference for specific scaffold geometries like triangular grids,^[^ ^52^, ^53^ ^]^ were employed for this study to promote cell spreading conducive to proliferation. Live/dead results revealed significantly improved cell performance on FDM‐reinforced scaffolds compared to unreinforced ones (Figure 4b and Figure S5b, Supporting Information). Closer examination (Figure 4c) using the inherently fluorescing PCL in the DAPI channel demonstrated that the form of the reinforced scaffolds was consistently maintained, remaining taut and significantly less prone to deformation. In contrast, the unreinforced scaffolds exhibited entanglement, collapse, and damage to pore size and geometry, which is visibly different from the ordered triangular shape shown to benefit cellular wellbeing^[^ ^53^, ^54^ ^]^ It has also been shown that cells do not adhere solely to a single fiber but can bridge certain distances from one fiber to another. Therefore, even slight deformations can lead to overstretching and damage to the cell membrane. These influences can affect overall vitality as well as the adhesion and proliferation behavior of the cells. The reinforcements not only enhanced handleability but also exerted a significant impact on cell culture outcomes. Consequently, FDM reinforcements prove beneficial for handling across various scenarios, ensuring more consistent and reproducible results. By maintaining scaffolds in the desired shape, these reinforcements contribute to reducing artifacts, errors, and the production of unusable, unrepresentative scaffolds for evaluation post‐experiment.

#### 2.4.2 Bioreactor Application

**Figure 5:**
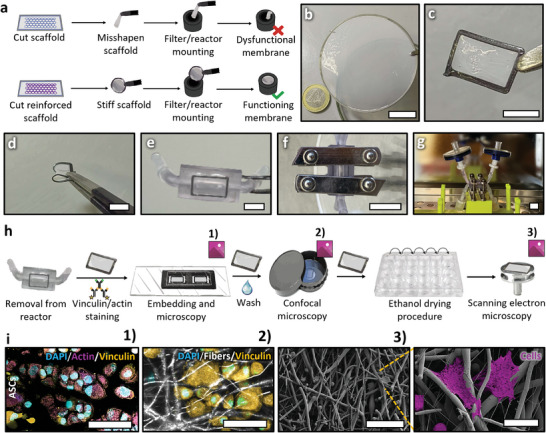
Improved scaffolds in a complex application‐oriented setting. a) Schematic showing the difference of mounting a scaffold without or with reinforcement rings to a bioreactor or filtration setup. b) A 7 cm diameter wetted electrospun PCL/gelatin membrane reinforced by a PLA ring, suspended in air. c) A square random electrospun PCL membrane scaffold used for a bioreactor experiment, with a soft TPU reinforcement frame, which can also function as a seal. d) The membrane from C, folded to show the TPU flexibility. e) The membrane mounted onto one side of a two‐chamber bioreactor. f) The two assembled bioreactor halves. g) The entire bioreactor setup mounted on a holder. h) Illustration showing the processes endured by the membrane post‐removal from the bioreactor after culturing. This involved primary/secondary anti‐vinculin/actin antibody staining, embedding in a microscopy chamber in Mowiol for microscopy or long‐term storage 1), subsequent washing out of the Mowiol and acquisition suspended in PBS using confocal microscopy to image the fibers and cells 2), followed by washing and the ethanol drying procedure for SEM imaging 3), all being conducted with the same sample. i) The acquired images from the processing shown in h labeled with 1, 2 and 3. Scale bar b) 2 cm, c,e,f) 6 mm and in i) 1–2) 50 µm 3) 80 µm and 20 µm on magnification.

In applications like bioreactors,^[^ ^28^, ^55^ ^]^ spheroid culture,^[^ ^56^ ^]^ or filtration, FDM‐reinforced membranes outperform their unreinforced counterparts by maintaining tautness and uniformity when mounted into reactors (**Figure** **5**a). Even large membranes, such as those made of a PCL/gelatin blend for lung models (Figure 5b), could be stabilized.^[^ ^28^ ^]^ The variable shape of the reinforcement (Figure 2c) allows a customized fit, and soft materials like TPU A60 can serve as seals for bioreactors (Figure 2d–f).

To showcase enhanced handling and imaging capabilities, an FDM‐reinforced electrospun random PCL membrane was incorporated into a bioreactor (Figure 5c–g). After a 3 d culture with Adipose‐derived stem cells (ASCs), the membrane was extracted, fixed, and stained for actin and vinculin (Figure 5h). Subsequently, it was embedded in Mowiol for long‐term storage and microscopy in a 3D printed chamber (Figure 5i1). Following imaging, the sample was withdrawn, suspended in a confocal dish in PBS, and imaged again for fibers and cells (Figure 5h,i2). The reinforcements prevented folding and deformation during dish movement, enhancing imaging quality. Post‐imaging, any Mowiol residues were washed out, and the sample was prepared for SEM imaging using an ethanol drying procedure (Figure 5h,i3). The reinforcements facilitated seamless transfers and drying without surface contact, preventing sticking. Throughout the entire process, the integrity of both cells and the membrane, intentionally selected for its challenge in conventional handling, remained preserved. Cells on the membrane maintained a rounded shape, preventing the formation of focal adhesions observed in the treated glass control (Figure S6, Supporting Information). Consequently, the FDM‐reinforced membrane mitigated detachment issues commonly encountered with fragile fibers and weakly adhering cells, even after various handling and deformation steps (Figure 5i3).

## 3 Discussion

Nano‐ and submicro‐fibers hold immense significance across various applications, with thousands of annual publications dedicated solely to electrospinning. This study addresses the pressing need for scalable and standardized methods to improve accessibility to these promising materials for both laboratory research and industrial production. We have developed a scalable procedure using readily available and cost‐effective technologies, enhancing consistency, production capacity, and time efficiency while also freeing up personnel resources. The new method involves sacrificial coating of the base substrate with PVA and reinforcing scaffold edges using FDM 3D printing with common biocompatible polymers. This combined approach facilitates easy detachment of scaffolds from the collector system, ensuring improved adhesion with the incorporation of glycerin in the PVA coating. Laser cutting and FDM 3D printing create scaffolds that are effortlessly removable and reinforced, maintaining tautness and preventing sample collapse or deformation when wetted during handling. Experimental assessments demonstrate notable improvements in cell culture applications, enhancing handleability, speed, and reliability of experiments, while preserving scaffold structure and shape.

Furthermore, this system proves advantageous in bioreactors or filter‐like applications, offering ease of application and post‐processing with size and shape‐tailorable FDM reinforcements. Soft plastics like TPU in FDM printing introduce bifunctional reinforcements, retaining scaffold shape and acting as a seal for reactor chambers. Moreover, the method allows for the design of geometrically, functionally, and physiologically customized membranes for personalized medicine, offering significant clinical benefits. The availability of standardized specimens in high throughput further simplifies the post‐treatment and functionalization of the membranes, for example, as drug release systems, potentially increasing therapeutic intervention success and cost efficiency. By integrating biocompatible, water‐soluble reinforcements, these systems have the potential to significantly impact diverse medical fields including dermatology, ophthalmology, neurodegenerative diseases, transplantation medicine, and immunology in the future. Their excellent applicability positions them as a promising bench‐to‐bedside strategy within the clinical realm.

In summary, the method presented here enhances the availability of reproducible electrospun and electrowritten substrates in biofabrication and tissue engineering. It provides a cost‐effective and easily implementable solution, resulting in significant improvements in cell culture and post‐culture handling. This versatile approach allows for the customization of scaffolds to meet diverse requirements without sacrificing time efficiency or reproducibility, thereby fostering collaboration and standardization across laboratories. Thus, this work contributes to overcoming crucial bottlenecks in harnessing the huge potential of these fiber materials.

## 4 Experimental Section

### PVA Coating

Polished steel plates or the rougher sides of aluminum foils, were cleaned with isopropanol, left to dry and then coated with a thin coat (ca. 0.03 mL cm^−2^) of a 100 mg mL^−1^ solution of poly(vinyl alcohol) (PVA Mw 30–70 kDa, Merck KGaA, Darmstadt, Germany) in water. The coating was done by pouring the solution onto the metal plate, doctor blading with a glass slide, and left to dry at RT. When greater adhesion to the PVA film was desired, up to 11% (v/v) glycerin (Carl Roth, Karlsruhe, Germany) was added to the PVA solution before casting.

### Electrospinning of PCL Membranes

The PCL fiber membranes were produced by using a voltage difference of 9.5 kV, which was applied onto a 20 G needle (Microlance BD, New Jersey, USA). 1000 µL of the 24% w/v polycaprolactone (45 kDa, Sigma Aldrich, MO, USA) in 99% pure 1,1,1,3,3,3‐hexafluoro‐2‐propanol (HFIP, abcr GmbH, Germany) solution were spun at a rate of 3 mL h^−1^ and a 17 cm distance between needle and collector. The grounded rotating drum collector (Ø 94 mm) was rotated at the speed of 1600 rpm for aligned (ca. 15 µm thickness) and 100 rpm for random (ca. 18 µm thickness) membranes. The membranes were spun onto an aluminum foil thinly coated with polyvinyl alcohol (PVA 30–70 kDa, Merck KGaA, Darmstadt, Germany) that was attached to the collector. The collected fiber membranes were submersed into a mixture of 70% ethanol (v/v) and soaked for ca. 1 min. The membranes were then, transferred using tweezers, washed thrice in H_2_O, dipped in 100% ethanol and dried.

### Electrospun PCL/Gelatin Membranes

8% w/v PCL (Mw 80 kDa Merck KGaA, Darmstadt, Germany) and gelatin 2% w/v (type A from porcine skin, Merck KGaA, Darmstadt, Germany), were dissolved in a solvent mixture composed of 1,1,1,3,3,3‐hexafluoro‐2‐propanol (HFIP)/formic acid (FA) (both from Sigma‐Aldrich, HFIP:FA; 9:1 v/v). The solution was electrospun using a blunt 27G needle and the solution extruded at the speed of 0.3 mL h^−1^, with a voltage of 15 kV applied to the needle. The grounded rotating drum collector (Ø 94 mm) was rotated at the speed of 100 rpm resulting in a membrane ca. 1 µm thick. The membranes were spun onto an aluminum foil thinly coated with polyvinyl alcohol (PVA 30–70 kDa, Merck KGaA, Darmstadt, Germany) that was attached to the collector.

### Electrospinning of PLA Membranes

A volume of 1.5 mL of a 2% Poly‐L‐Lactic Acid Mw 650 kDa (PLLA) (PL65 Purasorb, Netherlands) in HFIP solution was electrospun with a 27G nozzle, a voltage of 12 kV and a 15 cm distance from a rotating Ø 7 mm collector spinning at 100 rpm, to which, as previously mentioned, a PVA coated foil was attached. The collected fiber membranes (ca. 7 µm thickness) were submersed into 70% ethanol (v/v) and soaked for ca. 1 min. The membranes were then, transferred using tweezers, washed thrice in 70% ethanol, and dried.

### Production of PCL MEW Scaffolds

PCL box and triangular scaffolds were produced using melt electrowriting (MEW). The printing was conducted at a room temperature of 20 °C with 40% humidity, a PCL (Purac PC12, Corbion, Amsterdam, the Netherlands) melt temperature of 95 °C, a pressure of 1 bar and a print bed movement rate of 1000 mm min^−1^ onto a grounded steel build plate covered by a thin water‐soluble poly(vinyl alcohol) (PVA, 30–70 kDa, Merck KGaA, Darmstadt, Germany) coating a 2.5 kV voltage difference applied over the 30 G needle (Nordson EFD, Ebensfeld, Germany), at a printing distance of 1.4 mm. This yielded fiber diameters of 3 µm, with the scaffolds being printed for 10 layers in each direction. The angles of the box structure were 90° with 20 layers on the crossing points (60 µm total height), whilst the triangular structure was generated by overlapping the box shape grids at a 45° angle, resulting in a mix of scaffold pore angles of 90°, 45° and 135°, with 20 layers on the crossing points (60 µm total height). Before application the PVA was dissolved using 70% ethanol, by pouring it directly onto the metal plate, the scaffolds were removed using tweezers after the polymer coating had dissolved, washed in 70% ethanol and dried.

The thicker electrowritten PCL scaffolds were produced using similar parameters, except that a 25G needle (Nordson EFD, Ebensfeld, Germany), a pressure of 2 bar and a speed of 500 mm s ^−1^ were used, resulting in fiber diameters of 10 µm. Ten layers were printed in each direction, resulting in a scaffold height of ca. 100 µm or up to 200 µm on the crossing points of the layers.

### Laser Cutting

The scaffolds were laser cut (Rayjet, Trotec, Plymouth USA) to the appropriate well size. The speed and intensity of the laser were varied to achieve a complete separation of the remaining scaffold.

### FDM Printing onto Scaffolds

The scaffolds were reinforced with polylactic acid (PLA, Form Futura, Amsterdam, Netherlands), polyvinyl alcohol (PVA) (Form Futura, Amsterdam, Netherlands), or polycaprolactone (PCL) (Facilan Ortho, 3D4makers, Haarlem, The Netherlands) using a 0.4 mm nozzle, a layer height of 0.28 mm, a print speed of 5–20 mm s^−1^, a 10 mm retraction distance and 80 mm s^−1^ retraction speed, without a heated print bed and the nozzle temperatures of 180 °C, 190 °C, and 130 °C for the different polymers respectively. Thermoplastic polyurethane (TPU) (FilaFlex 60A, Recreus, Elda, Spain) was printed using the same conditions, except that the nozzle temperature was 210 °C and the retraction was disabled.

### Tensile Testing

The testing of the different reinforcement rings and scaffold was performed with a universal testing machine (Z010, Zwick Roell, Ulm, Germany) with a 100 N load cell. The samples were stretched with a velocity of 10 mm min^−1^ mounted between two clamps. The upper force limit was set to 95 N. The force dependent on the stretch was measured and evaluated.

### Hanging Test Wet/Dry

The hanging test was performed using a 3D printed construct into the beak of which the edges of the mesh were fastened. A background showing the different angles from 0° to 90° in steps of 10° was placed. The scaffolds were tested dry and wet, whereby the wet scaffolds were wetted by letting these absorb the liquid they could take up. A photograph was taken at a perpendicular height to the mesh. Triplicates were performed for each experiment and the angle of the hanging scaffold was determined.

### Projector Calibration

To calibrate the projector (YABER V5, YABER, Austin, USA) to the build plate of the FDM printer (modified Ender 3 V2, Creality, Shenzen China), a calibration print was conducted, upon which the projection was calibrated. Thereafter the calibrated projection was used to calibrate the laser cut substrates to the correct position, where the printed strut would then be deposited.

### FTIR Measurements

The samples were measured dry using a Nicolet iS10 with smart iTR diamond ATR (attenuated total reflectance, Thermo Fisher Scientific, Waltham, USA).

### Scanning Electron Microscopy (SEM)

The samples were analyzed using a SEM device (Crossbeam CB 340 SEM, Carl Zeiss) after being sputter coated (EM ACE600, Leica) with 4 nm of platinum.

### Ethanol Drying Procedure

The already fixed cell samples were transferred to PBS and thoroughly washed. Afterwards, these were incubated in 70%, 90% and 100% ethanol, twice for 10 min for each respective step. Thereafter the samples were incubated in hexamethyldisilazan (HMDS) (Merck KGaA, Darmstadt, Germany) twice for 10 min and subsequently left to dry.

### U87 Culture and Seeding

U‐87 MG (ATCC HTB‐14, LGC Standards GmbH, Germany) were cultured in Dulbecco's modified Eagle medium (DMEM) (41966‐029, Gibco, MA, USA) supplemented with 10% FCS (10270‐106 Life Technologies, MA, USA) and 10000 U mL^−1^ pen/strep (15140‐122 Life Technologies, MA, USA). Cells were split twice per week.

Scaffolds were sterilized with 70% ethanol and were placed 15–30 min under UV light. Afterwards scaffolds were washed three times with ddH_2_O and once with PBS. 3 cm dishes with four 93 mm^2^ inner rings (627170, Greiner, Greiner Bio‐One, Kremsmünster, Austria) were used to place the scaffolds and add 50 µl of full media. Finally, scaffolds were incubated for 30 min at 37 °C with 5% CO_2_, thereafter the cells were added at a concentration of 10 000 cells per well and further incubated.

### Ethical Statement

Experiments were approved by the local veterinary authority (Veterinäramt der Stadt Würzburg, Germany) and the Ethics Committee of Animal Experiments, i.e., Regierung von Unterfranken, Würzburg, Germany (license no.: FBVVL 568/200‐324/13).

### Astrocytes Isolation and Culture

CD‐1 pups (P0‐P1) were used to isolate primary astrocytes. After extracting the brains, cortices were dissected and collected in ice‐cold phosphate‐buffered saline (PBS). Following a brief homogenization and filtration through a 70 µm cell strainer (542070, Greiner Bio‐One, Kremsmünster, Austria), cells were centrifuged (10 min, 1400 rpm), resuspended and seeded in 6 cm dishes with 5 mL of DMEM supplemented with 10% fetal calf serum, 2 × 10^−3^ m GlutaMAX, 1 × 10^−3^ m sodium pyruvate, and 50 U mL^−1^ penicillin/streptomycin (15140‐122 Life Technologies, MA, USA). Astrocytes grew under standard conditions at 37 °C with 5% CO_2_. Cells were washed with PBS and medium was exchanged 3–4 days after seeding. After seven days, cells were detached and counted. 150000 astrocytes in suspension were pipetted on top of each scaffold. An O‐metal ring was used to fix the scaffolds. Afterward 3 mL of supplemented DMEM medium was added.

### Adipose‐Derived Stem Cell (ASCs) Culture and Seeding

Cells were centrifuged (5 min, 1200 rpm), resuspended and 15.000 cells were seeded on the electrospun membranes and on control glass slides in well plates with 5 mL of DMEM F‐12 (1:1) supplemented with 200 mm GlutaMAX, 100 U mL^−1^ penicillin/streptomycin (Thermo Fisher Scientific, Waltham, MA), 10% fetal calf serum, basic fibroblasts growth factor (FGF) and 50 µg mL^−1^ ascorbic acid (Sigma‐Aldrich, Germany). ASC grew under standard conditions at 37 °C with 5% CO_2_ atmosphere for three days. Medium was exchanged one day after seeding and every day after.

### Immunocytochemistry

For adipocytes the random electrospun PCL membranes were removed from the reactor by wetting the sides of the membrane outside of the main chamber with PBS Astrocyte/Adipocyte were washed once with PBS (pH 7.4) and fixed for 20 min with a 2% paraformaldehyde (PFA) solution or 3.7% gluteraldehyde. Following fixation, astrocytes were permeabilized and blocked with 5% normal goat serum (NGS) with 0.2% Triton‐X 100 in PBS for 30 min. Adipocytes were treated with 0.1% TritonX‐100 in PBS for 5 min and blocked with 5% BSA in PBS for 30 min at room temperature. Astrocytes were incubated with ActinGreen 488 ReadyProbes Reagent (R37110 Invitrogen, Carlsbad, CA) in blocking solution for 1 h. Finally, scaffolds were mounted with ProLong Glass Antifade Mountant containing Hoechst 33 342 (Thermo Fisher Scientific, Waltham, MA) on glass slides. Adipocytes were washed with PBS and incubated with primary antibody anti‐vinculin (1:50; V4505 Sigma Aldrich, Germany) for 1 h followed by secondary antibody incubation goat anti‐rabbit‐Cy3 (1:500, 111‐165‐003 Dianova, Hamburg, Germany). In the same step, ActinGreen 488 readyProbes (1:50 R37110 Invitrogen, Carlsbad, CA) reagent staining was included. Cells were stained with DAPI (1:5000, D3571 Invitrogen, Canada) for 10 min and mounted on glass slides with Mowiol 4–88 (81381‐50G Sigma Aldrich, Germany). The embedding was done in a FDM 3D printed chamber, slightly higher than the reinforced scaffold, and glued to a glass slide using nail lacquer, while the top was also sealed with glass and lacquer. This was done to easily embed the whole scaffolds and remove all bubbles.

### Live Dead Staining of U87 Cells and Primary Astrocytes

The staining was performed at day 1 and day 7 post‐seeding at 21 °C for 20 min with Calcein‐AM (2 × 10^−6^ m, green/living cells; Thermo Fisher Scientific, Waltham, MA) and ethidium homodimer (2 × 10^−6^ m, red/dead cells; Sigma‐Aldrich, St. Louis, MO) diluted in PBS and incubated for 20 min.

### Confocal Microscopy and Image Acquisition

Samples were imaged using an inverted Olympus IX81 microscope equipped with an Olympus FV1000 confocal laser scanning system, a FVD10 SPD spectral detector, and diode lasers of 405 nm (DAPI), 473 nm (Alexa488) and 559 nm (Cy3) (Olympus, Tokyo, Japan). All images shown were acquired using an Olympus UPLSAPO 10× (air, numerical aperture 0.4) or Olympus UPLFLN 40x (oil, numerical aperture: 1.3) and were processed using ImageJ/Fiji 1 and Imaris 7.7.2 (Oxford Instrumentals, Abingdon, UK). For cell viability, z‐stacks of about 2–3.52 µm step size throughout each sample were acquired. Imaris was used for 3D reconstruction, video generation and reconstruction of the z‐stack images to quantitatively analyze live and dead cell numbers The Spots function was used to determine the live/dead ratio. 5 image stacks per experimental condition were analyzed (*N* = 3). Dynamic range adjustments and projections were done with ImageJ/Fiji Software.

### Statistical Analysis

GraphPad Prism 8.3.0 (Graphpad Software, San Diego, CA, USA) was used to calculate mean values, standard deviation (SD), standard error of the mean (SEM), and values for statistical significance. Statistical significance was estimated *p < 0.05 using two‐way ANOVA.

## Conflict of Interest

The authors declare no conflict of interest.

## Supporting information



Supporting Information

## Data Availability

The data that support the findings of this study are available from the corresponding author upon reasonable request.
