# Supplementary material for: Streamlining the Highly Reproducible Fabrication of Fibrous Biomedical Specimens toward Standardization and High Throughput
Source: Adv Healthc Mater. 2024 Dec 15;14(4):2402527. doi: 10.1002/adhm.202402527 (PMC11804836; doi:10.1002/adhm.202402527)
Supplement: Supplementary file 1 — Supporting Information [file ADHM-14-0-s001.docx]

**Streamlining the Highly Reproducible Fabrication of Fibrous Biomedical Specimens towards Standardization and High Throughput**

**Supplement**

*
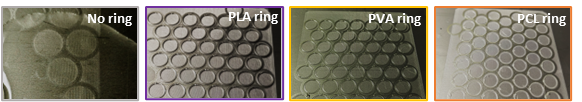
*

**Fig. S1:** Zoomed out pictures of laser cut and FDM reinforced MEW scaffolds with rings made of PLA, PVA and PCL.


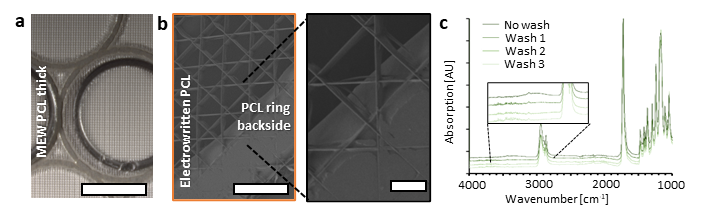


**Fig. S2: (a)** Thick MEW scaffold reinforced with PLA rings. (**b**) The backside of an electrowritten scaffold melted onto the PCL reinforcement ring. (**c**) FTIR spectra showing the removal of PVA from electrospun scaffolds after several washing steps in 70 % ethanol. Scale bar in (**a**) 5 mm, in magnification 100 µm and 400 µm in (**b**).


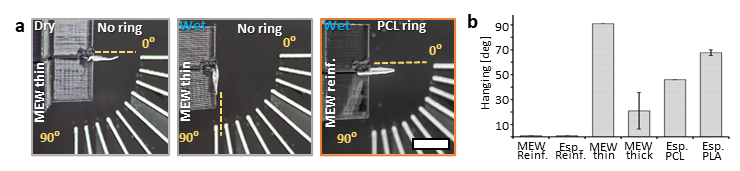


**Fig. S3:** (**a**) Exemplary pictures of a semi-quantitative hanging angle test, conducted with dry and wet scaffolds, with and without reinforcement rings, showing the ability of the scaffolds to remain stable when dry and wetted, in this case using thin MEW scaffolds. (**b**) The hanging angle test results under wet conditions shown for scaffolds. Scale bar (**a)** 1 cm.


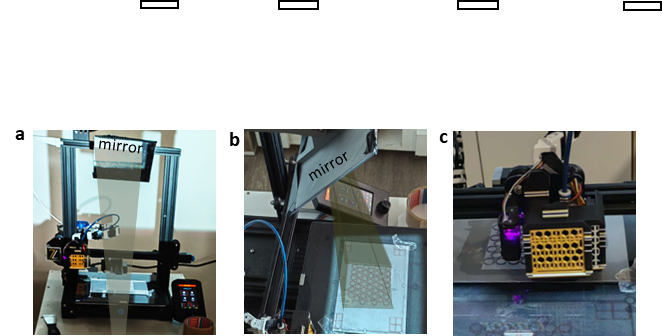


**Fig. S4**: (**a**) Actual setup of the FDM printing calibration using a projector, an image looking at the printer from behind the projector (projection is sketched in color). (**b**) An image from the side of the printer showing the substrate on the metal plate and projected pattern aligned. (**c**) The printer printing atop the scaffold.


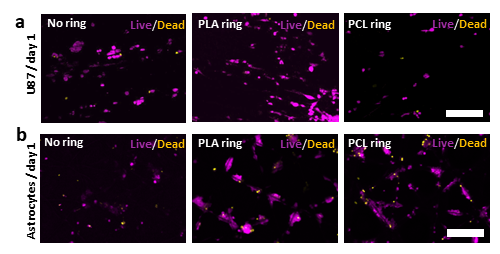


**Fig. S5:** (a) Live/dead of U87 on aligned electrospun PCL membranes without and with PLA/PCL reinforcement rings on day 1. (b) Astrocytes live/dead on MEW scaffolds without and with PLA/PCL reinforcement rings on day 1. Scale bar (a) 100 µm and (b) 200 µm.


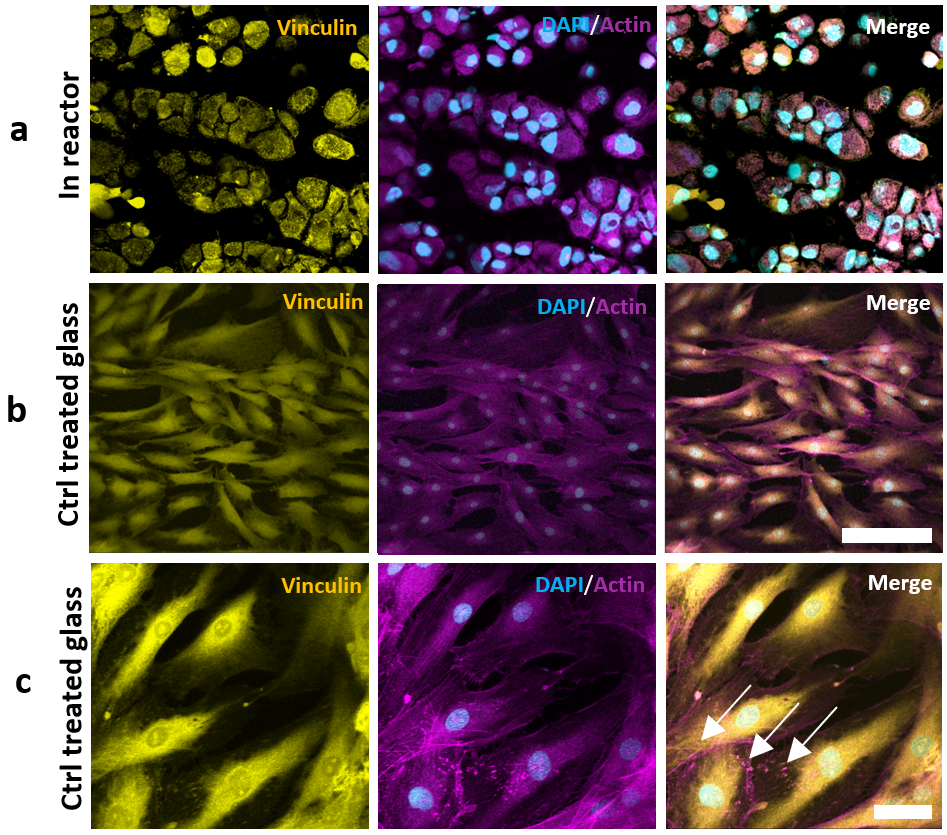


**Fig. S6:** (a) Vinculin/Actin staining of ASCs on the random electrospun PCL membrane in the bioreactor. (b) The same staining on a treated glass control. (c) Magnified images of locations on the glass slide showing focal adhesions. Scale bar (a, b) 50 µm and (c) 10 µm.
